# Lesion Size- and Location-Dependent Recruitment of Contralesional Thalamus and Motor Cortex Facilitates Recovery after Stroke in Mice

**DOI:** 10.1007/s12975-020-00802-3

**Published:** 2020-03-12

**Authors:** Markus Aswendt, Niklas Pallast, Frederique Wieters, Mayan Baues, Mathias Hoehn, Gereon R Fink

**Affiliations:** 1 Department of Neurology, Faculty of Medicine, University of Cologne and University Hospital Cologne, Kerpener Strasse, 62 50937 Cologne, Germany; 2 Cognitive Neuroscience, Research Center Juelich, Institute of Neuroscience and Medicine (INM-3), Juelich, Germany; 3 Department of Radiology, Leiden University Medical Center, Leiden, Netherlands

**Keywords:** Stroke, Photothrombosis, DTI, Fiber tracking, Recovery of function, Structural connectivity

## Abstract

Brain lesions caused by cerebral ischemia or hemorrhage lead to a local breakdown of energy homeostasis followed by irreversible cell death and long-term impairment. Importantly, local brain lesions also generate remote functional and structural disturbances, which contribute to the behavioral deficit but also impact the recovery of function. While spontaneous recovery has been associated with endogenous repair mechanisms at the vascular, neural, and immune cell levels, the impact of structural plasticity on sensory-motor dysfunction and recovery thereof remains to be elucidated by longitudinal imaging in a mouse model. Here, we applied behavioral assessments, in vivo fiber tracking, and histological validation in a photothrombotic stroke mouse model. Atlas-based whole-brain structural connectivity analysis and ex vivo histology revealed secondary neurodegeneration in the ipsilesional brain areas, mostly in the dorsal sensorimotor area of the thalamus. Furthermore, we describe for the first time a lesion size-dependent increase in structural connectivity between the contralesional primary motor cortex and thalamus with the ipsilesional cortex. The involvement of the contralesional hemisphere was associated with improved functional recovery relative to lesion size. This study highlights the importance of in vivo fiber tracking and the role of the contralesional hemisphere during spontaneous functional improvement as a potential novel stroke biomarker and therapeutic targets.

**Electronic supplementary material:**

The online version of this article (10.1007/s12975-020-00802-3) contains supplementary material, which is available to authorized users.

## Introduction

Stroke is a leading cause of death and disability worldwide with to date limited treatment options both in the acute and chronic phase [1]. Previously, the majority of experimental studies investigated the pathophysiology of stroke, and potential treatments focused on neuroprotection with infarct size as a final readout. However, none of these studies was translated successfully into the clinic [2] resulting in an increasing interest of the neural processes supporting recovery of function. In stroke patients, the relationship between lesion location, size, and functional recovery remains controversial and challenging to compare across studies [3, 4]. The lesion location within a particular brain network and the extent of damage to ascending or descending pathways all contribute to the stroke-induced deficit and are likely related to the long-term outcome [5, 6]. In animal models, structural plasticity in the regions close to the ischemic lesion as well as homotopic areas of the contralesional hemisphere have been assessed mostly by histological studies of axonal sprouting and in vivo two-photon microscopy of dendritic spine dynamics [7, 8]. In stark contrast to investigations of recovery of function in humans in experimental stroke models, remote changes after ischemic infarcts, such as secondary neurodegeneration, and their functional significance remains understudied [9, 10].

Changes in structural connectivity can be assessed using diffusion-weighted MRI (DWI) and T2-weighted MRI (T2WI), which detect the evolving injury and the presence or absence of edema, respectively [11]. DWI is sensitive to water movements dependent on tissue type, integrity, and presence of barriers. Diffusion tensor imaging (DTI) is a specific analysis scheme that allows to measure diffusion along multiple orientations and to infer the three-dimensional displacement of tissue water. White matter damage measured by DTI has been correlated with brain recovery in human and animal studies [12, 13]. However, there have been only single applications of in vivo DTI and in vivo fiber tracking in animal models of stroke with no conclusive correlation to neither the behavioral deficit nor the outcome [14, 15]. Here, we hypothesized that depending on the stroke size and location, different structurally connected but remote regions undergo secondary neurodegeneration and that distant but functionally connected brain regions are involved in spontaneous recovery of function. We present longitudinal in vivo fiber tracking data over 4 weeks from mice with local cortical brain lesions of different sizes. An atlas-based post-processing regime was applied to study DTI-based structural connectivity changes in ipsi- and contralesional cortical and subcortical fiber tracts. Spontaneous recovery was measured by a combination of behavior tests and correlated with the lesion and changes in fiber density.

## Results

### Characterization of Lesion Size

**Fig. 1:**
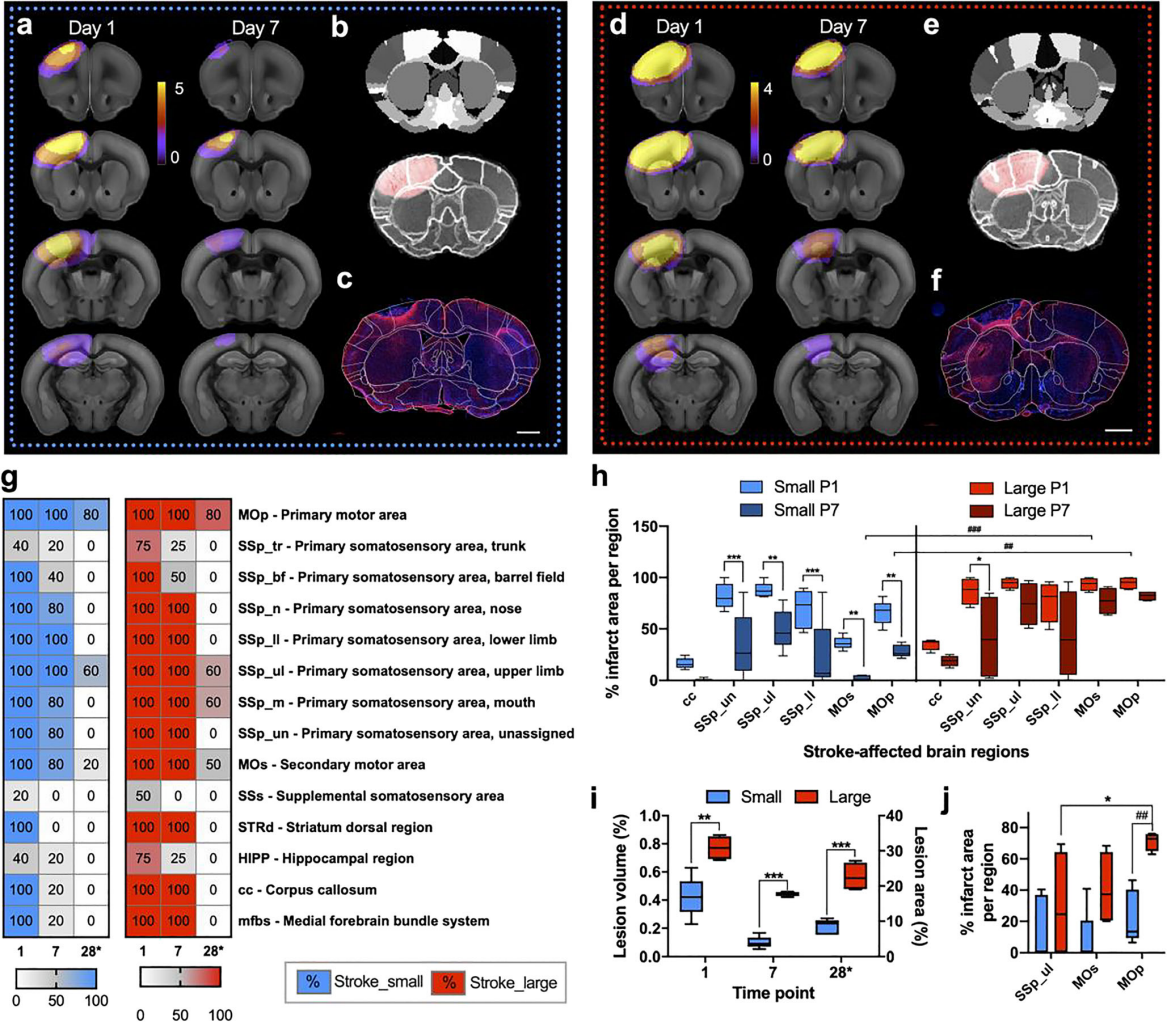
Quantification of lesion size and location for the two stroke protocols aiming for small (blue) and large (red) infarct, respectively, in the somatosensory and motor cortex. **a**, **d** Individual stroke masks based on T2WI were averaged across all mice and plotted as incidence maps and overlay with the Allen Mouse Brain Reference Atlas (ARA). Image sections selected to show the maximal extent of ischemic injury at day 1 and 7 after photothrombosis resulting in small (**a**) and large (**d**) strokes. **b**, **e** A multi-step registration of the ARA with the T2WI achieved a detailed atlas-based lesion analysis was achieved by a multi-step registration of the ARA with the T2WI. Less brain distortion due to the space occupying effect of the lesion (red area) was visible in small (**c**) compared with large strokes (**f**). **c**, **f** A landmark-based registration was used to register selected ARA plates with stitched whole mouse brain section histology (GFAP, red; DAPI, blue; scale bar 1 mm) shown here as an overlay with atlas labels for the small (**c**) and large (**f**) strokes groups. **g** Lesion mapping from in vivo and ex vivo imaging summarized as heat maps for small (blue) and large (red) strokes, where the color code indicates the percentage of mice in which a specific brain atlas label was inside the stroke mask at days 1/7 (MRI) and 28* (histology), selected regions only, for the full list see Supplementary Material Fig. 1a. **h** Calculation of percent infarct per selected brain region corpus callosum (cc), primary somatosensory unassigned (un)/upper limb (ul)/lower limb (ll), and primary and secondary motor area (MOs and MOp). **i** Quantitative lesion size analysis based on the T2-weighted MRI (left *y*-axis) and GFAP histology (right *y*-axis), respectively. **j** Calculation of percent infarct per selected brain region based on histology for GFAP. Significant differences between the groups at the same time points (*) and at different time points (^#^) are shown as */^#^*p* ≤ 0.05, **/^##^*p* ≤ 0.01, and ***/^###^*p* ≤ 0.001, respectively

By applying two different protocols of photothrombosis, ischemic cortical lesions of different sizes and locations were induced in adult C57Bl/6J mice. The lesion size and location were determined by T2-weighted MRI (T2WI) at 1 and 7 days post stroke. A 3D stroke mask was used to calculate individual stroke volumes. The lesion size and location at 4 weeks post stroke were quantitatively validated by immunohistochemistry of GFAP related to astrogliosis and the glial scar. The MRI and microscopy data were co-registered with a mouse brain atlas with MRI-voxel size-adjusted parental brain regions derived from the Allen Brain Reference Atlas (ARA) [16]. Two distinct stroke groups were defined based on the lesion location and size: one group with small (Fig. 1a–c) and one group with large (Fig. 1d–f) lesions.

Quantitative lesion mapping (Fig. 1g**,** complete list in Supplementary Material, Fig. 1a) and *t* test comparison revealed that in the small strokes group, the ischemic lesion was less frontal. Besides, specific brain regions were less often affected compared with the large strokes group (*p* = 0.013). From day one to seven, in the small strokes group, the number of brain regions affected was significantly smaller (*p* = 0.028). Specific brain regions such as the dorsal region of the striatum (STRd) and the anterior cingulate area (ACG) were inside the stroke mask at both time points in the large stroke group only. Compared with the brain regions marked as ischemic territory by histology at 4 weeks post stroke, in both groups, the number of affected brain regions was significantly reduced (*p* < 0.001). However, the initial difference between the groups in terms of how often a specific brain region was affected was no longer present at 4 weeks post stroke (*p* > 0.999). Between days one and seven, the overall MRI lesion volume was reduced by a factor of 1.7 in the large compared with 4.3 in the small strokes group (*p* = 0.004 and *p* < 0.001). The T2WI volume was significantly different at both evaluated time points, days one and seven, between the small and large strokes groups (*p* = 0.002 and *p* < 0.001). In line with the MRI, at 4 weeks post stroke, the lesion area determined with GFAP immunohistochemistry was 2.5 times larger in the large compared with the small strokes group (*p* < 0.001) (Fig. 1h). There was a strong correlation between the lesion sizes determined by MRI and histology (Supplementary Material, Fig. 1b). Not only the T2WI lesion size at day one was a reliable indicator of the final lesion size at day 28, but it was also possible to separate two groups according to the lesion size by hierarchical clustering. For the lesion size, the inter-cluster distance was 25 while the intra-cluster distance was below 2.5 (Ward-linkage). These two groups resemble precisely the two experimental groups of mice which received the different photothrombotic protocols, which underlines the non-arbitrary categorization into small and large strokes. For selected gray and white matter regions (cc, SSp-un/ul/ll, MOs, and MOp), we further quantified the percentage of infarct area per region to account for the fact that not the entire regions were covered by the ischemic lesion (Fig. 1h). Similar to the overall lesion size, the final percentage of infarct area covered in the MOp at 4 weeks post stroke was predicted by MRI at days one and seven, respectively (Supplementary Material, Fig. 1c). While in the small strokes group, the lesion part for all selected cortical regions decreased from days one to seven significantly (all *p* < 0.01); the large strokes decreased in space only close to the SSp_un (*p* = 0.019). Furthermore, in MOs and MOp, the percentage of infarct area was significantly different between small and large strokes on days 1 and 7, respectively. For example, the affected part of the MOp at day 7 was 2.47 ± 2.54% in the small strokes and 77.30 ± 13.57% in the large strokes (*p* < 0.001). The T2WI lesion volume and the number of affected brain regions were reduced within the first week (Fig. 1i, g). The consolidation of the lesion was expressed as the slope between the stroke-affected brain area percentage at days 1 and 7 (Supplementary Material, Fig. 1d**)**. Notably, the slope was different for the motor areas only (MOs: *p* = 0.009 and MOp: *p* = 0.003). In the chronic phase, i.e., 4 weeks after stroke, the lesion was consolidated in SSp_ul, MOs, and MOp with a preference for the MOp over SSp_ul (*p* = 0.036). Likewise, between the groups, there was only a significant difference in the percentage of infarct area per MOp region (*p* = 0.008) with 22.49 ± 16.97% for small and 71.09 ± 5.89% for large strokes, respectively (Fig. 1j). Inside the lesion mask, determined on the basis of the T2WI, the clinically-relevant diffusion measures molecular diffusion rate (MD), radial diffusivity (RD), and axial diffusivity (AD) were significantly different between the two groups only in the subacute phase at day 7 (*p* = 0.003, *p* = 0.030, *p* = 0.009) but not at later time points (Supplementary Material, Fig. 1e). The diffusion measure of axonal injury, i.e., AD, significantly increased over time without any difference between the groups (*p* < 0.001) while RD did not change at all. Membrane density and microstructural integrity as reflected in MD and fractional anisotropy (FA) diffusion measures increased from within the first week in the small strokes group (*p* = 0.001 and *p* = 0.004) only and not the large strokes group. From days 7 to 28, MD and FA increased in both groups (*p* < 0.001) until a similar level was reached.

### Lesion Size-Dependent Functional Deficit and Recovery

**Fig. 2:**
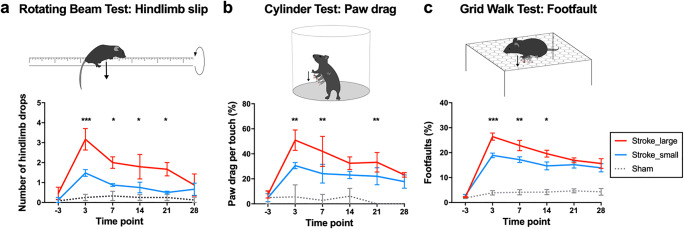
Stroke lesion size determines initial sensorimotor deficit but not recovery level in three different behavioral tests. **a** Number of hindlimb drops recorded for a walking task in which the mouse walks over a rotating beam towards the homecage on a platform. **b** Number of paw drags calculated as percentage per touch with the affected paw at the cylinder wall. **c** Number of foot faults with the affected paw calculated as percentage per total number of footsteps. Data in graphs are shown as mean ± sem. Significant differences between the groups at the same time points shown as *p ≤ 0.05, **p ≤ 0.01, and ***p ≤ 0.001, respectively

We assessed the sensorimotor skills before and after stroke using the following behavioral tests for the assessment of motor performance: rotating beam, cylinder, and grid walk, which probe spontaneous and voluntary locomotion, motor coordination, and grasping. Except for early time after stroke, mouse behavior and recovery after stroke were not different between groups based on the general measures of body weight and neurological deficit score, as well the less specific, and thus less sensitive analysis of paw touch (cylinder test) and speed and distance (rotating beam test). For example, the number of touches with the affected paw was different between the groups only at day 3 (*p* = 0.033) (Supplementary Material, Fig. 2). In contrast, in the more sensitive measures of hindlimb drop while walking over the beam, paw drags on the cylinder wall and foot faults on the grid (Fig. 2), the mice with the large strokes performed significantly worse early after stroke (day 3, *p* < 0.001). The stark differences in sensorimotor behavior were detected until day 14 (grid walk, *p* = 0.018) or 21 (rotating beam *p* = 0.023 and cylinder test *p* = 0.022). Importantly, in contrast to the lesion size that remained different between small and large strokes, we detected no significant differences in terms of sensorimotor behavior between the groups at 4 weeks after stroke in all measures.

We further correlated lesion size and location with the behavioral deficit at day three as well the functional outcome at day 28 (Supplementary Material Table 1**)**. The initial lesion size and the percentage of stroke-affected MOp, measured by MRI at day one after stroke, were correlated with the number of foot faults and hindlimb drops measured at day three after (*p* = 0.033/*p* = 0.043 and *p* = 0.023 and *p* = 0.010) but not 4 weeks after stroke. However, a large behavioral deficit related to a high number of foot faults or hindlimb drops at day three was positively correlated with a large remaining stroke lesion at day 28 (*p* = 0.001/*p* = 0.002). That correlation was independent of the percentage of affected MOp at day 28.

### Secondary Neurodegeneration and Reduced Structural Connectivity in Ipsilesional Thalamus

For the region-based analysis of whole-brain DTI connectivity data, anatomical connections described in the Allen Mouse Brain Connectivity Atlas were used as a reference. The sham animals served as control mice for which the reliability of DTI was verified by existing viral tracing [17]. All DTI data were positively correlated to the sensory-motor cortex-related part of the thalamus (DORSm) viral tracing data with a mean covariance *c* = 0.016 ± 0.004 and a mean linear correlation coefficient *ρ* (rho) = 0.430 ± 0.079 with *p* = 0.011. Ranked according to the largest viral projection density (which is in the injection zone of the DORsm) and the fiber density of the DTI (which is the DORsm itself), the two completely different measures result in a very similar distribution of connected regions (Supplementary Material Fig. 3).

**Fig. 3:**
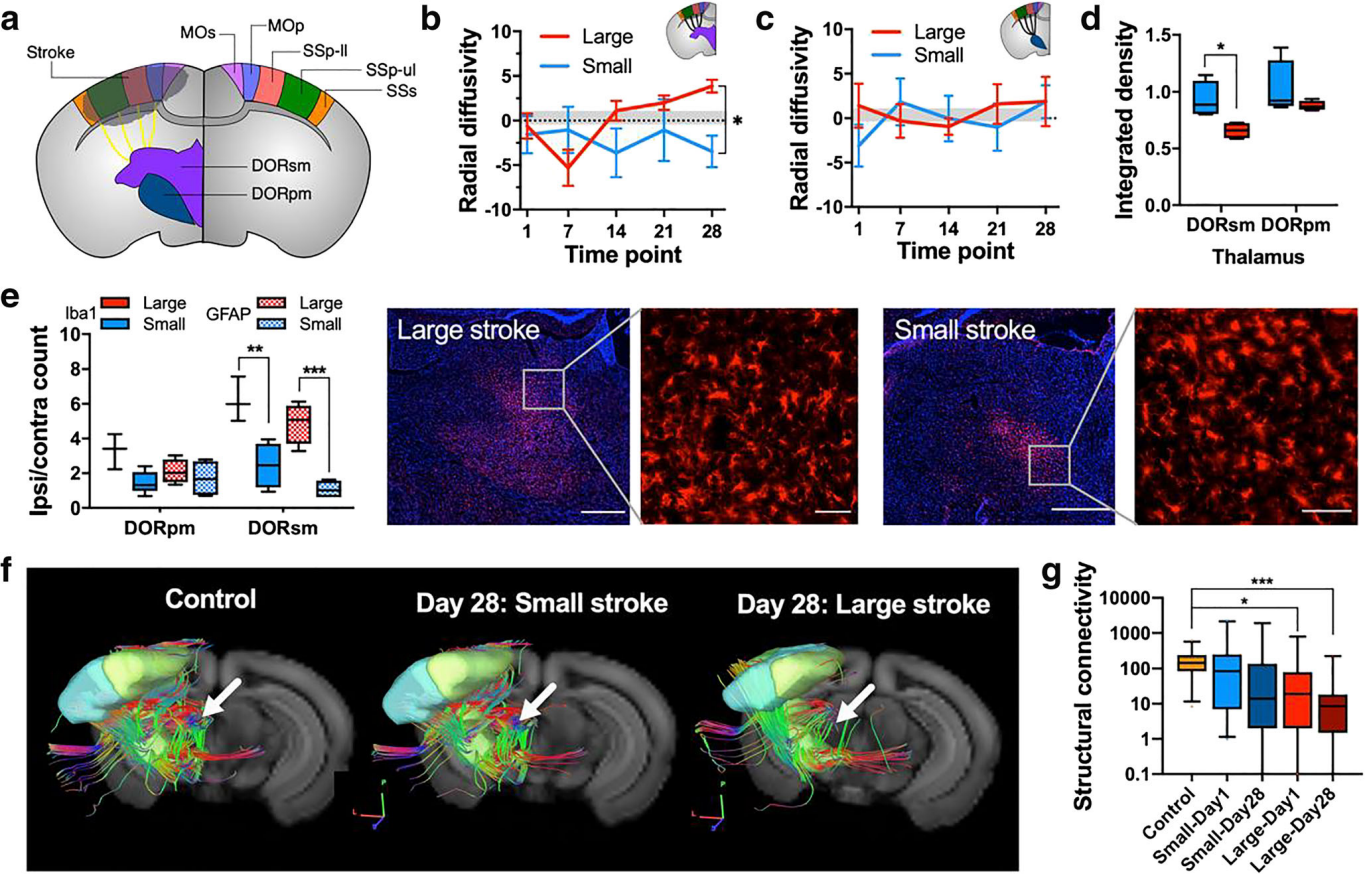
Lesion size-dependent secondary degeneration of thalamocortical fiber tracts. **a** Schematic drawing for the selected thalamocortical fiber tracts between the sensory-motor cortex (MOp, MOs, SSp-ll/−ul, and SSs), the related parts of the thalamus: DORsm and DORpm. **b–c** Longitudinal diffusion measures for the DORsm and DORpm region expressed as radial diffusivity (RD) [(ipsi-contra)/contra × 100%] for the large (red) and small (blue) strokes groups, respectively, and control values (gray). **d** Integrated density (which is the ratio ipsilateral/contralateral for the signal intensity normalized to the region size) for DORsm and DORpm regions on T2WI. **e** Counting results for Iba1^+^ and GFAP^+^ cells in the thalamus. Representative immunofluorescence microscopy of a coronal section of the thalamus (red, Iba1 immunostaining; blue, DAPI nuclei counterstain, scale bar 500 μm and 50 μm). **f** Representative in vivo fiber tracking results for the selected DORsm thalamocortical tract in controls and small/large strokes at day 28. **g** Differences in average transhemispheric connectivity for the selected contralesional MOp to ipsilesional MOs/MOp/SSp-ll/SSp-ul/SSs cortex pathway for large/small strokes at day one and 28 compared with control. Significant differences between the groups are shown as *p ≤ 0.05, **p ≤ 0.01, and ***p ≤ 0.001, respectively

We selected the ipsilesional thalamocortical pathway (Fig. 3a) with efferent connections from the two functional main components of the thalamus (according to the ARA ontology), DORsm, and the polymodal association related part of the thalamus (DORpm). The five top-level cortical target areas (MOp, MOs, SS-ll, SS-ul, SSs) were selected based on a calculation of the average viral tracing projection density from all experiments targeting the DORsm and DORpm area used for the ARA connectivity atlas [17]**.** The DORsm (Fig. 3b) and DORpm (Fig. 3c) showed a different development in the diffusion measure, radial diffusivity (RD), over time. Significant differences compared with the control group (see “Material and Methods” section about the MRI control group) were detected only for the DORsm and for the large strokes where RD values decreased on day 3 (*p* = 0.035) and increased above control level on day 28 (*p* = 0.020). A significant difference between the small and large strokes group was detected for the DORsm only (*p* = 0.008). The analysis of integrated signal intensity (ipsilesional normalized to contralesional) on T2WI for the registered DORsm and DORpm regions revealed a significant difference for the small vs. large strokes in the DORsm only (*p* = 0.023), which is a sign of the accumulation of iron (Fig. 3d). In this line, homologous brain sections at the thalamic level were stained for GFAP (astrogliosis) and Iba1 (microglia and macrophage activation/accumulation) (Fig. 3e). In the large strokes group, the number of GFAP^+^ and Iba1^+^ cells was increased in the DORsm but not the DORpm compared with the small strokes (*p* < 0.001 and *p* = 0.001). We concluded that the secondary neurodegeneration is mainly present in the DORsm part of the thalamus and therefore continued with the fiber tracking analysis of DORsm to the cortex. In line with the initially observed increase in RD in the large strokes and a separation of RD between small and large strokes at 4 weeks post stroke, we detected a reduced fiber density for thalamocortical connections in the large strokes compared with small and control group (Fig. 3f). Quantitative fiber tracking comparison of the acute (day 1) and chronic (day 28) phase after stroke showed a significantly decreased structural connectivity between the control and the large strokes group (*p* = 0.023 and *p* < 0.001) in the ipsilesional but not the healthy hemisphere (Fig. 3g). A detailed analysis of the individual ipsilesional thalamocortical pathways revealed the DORsm to SSp-ul and SSp-ll to contribute most significantly contributing to the overall loss in structural connectivity in the large strokes group (*p* < 0.001 and *p* = 0.005). The decreased structural connectivity due to secondary neurodegeneration in the DORSm to SSp-ll pathway of the large strokes group predicted well the number of hindlimb drops on the rotating beam at day 28 (*R*^2^ = 0.961, *p* = 0.020).

### Increased Structural Connectivity in the Contralesional Thalamocortical Pathway

**Fig. 4:**
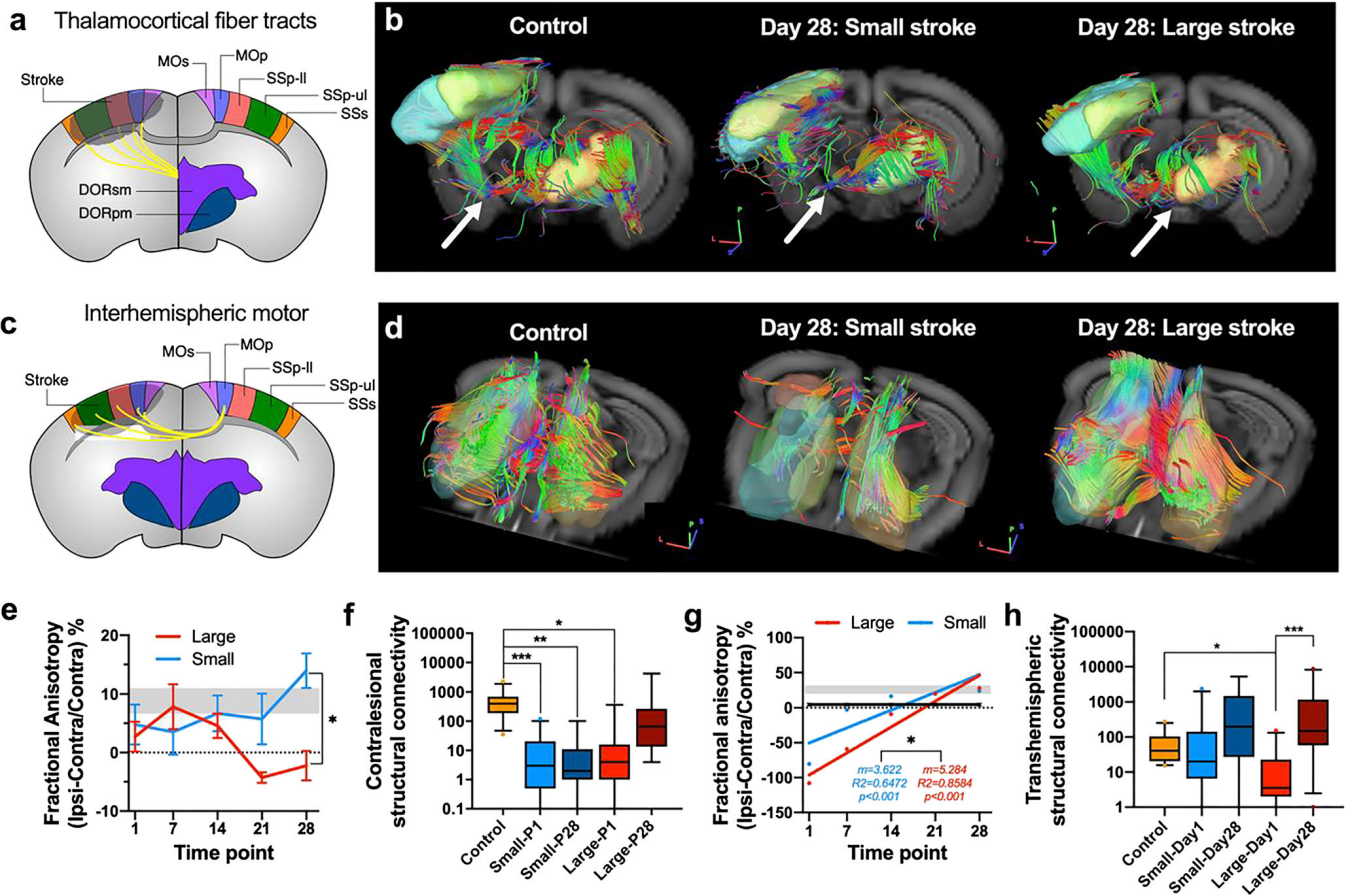
Lesion size-dependent increase in connectivity between contralesional (contra) primary motor cortex and ipsilesional (ipsi) sensorimotor cortex. **a** Selected thalamocortical fiber tracts between the sensory-motor cortex (MOp, MOs, SSp-ll, SSp-ul, SSs). The related parts of the thalamus: DORsm and DORpm. **b** Representative 3D visualization of fiber tracking results for the controls, small and large strokes groups (day 28). **c** Selected contra MOp to ipsi MOp, MOs, SSp-ll, SSp-ul, and SSs pathway for DTI analysis. **d** Representative in vivo fiber tracking results for the selected DORsm thalamocortical tract in controls and small/large strokes at day 28. Arrows pointing to differences in fiber density. **e** Longitudinal measures of FA for the DORsm and DORpm region [(ipsi-contra)/contra × 100%] for the large (red) and small (blue) strokes group and control values (gray). **f** Structural connectivity from contra DORsm to ipsi MOp, MOs, SSp-ul/ll, and SSs. **g** Linear regression analysis for the diffusion measure FA [(ipsi-contra)/contra × 100%] calculated for 5 measurements post stroke. **h** Differences in average transhemispheric connectivity for the selected contralesional MOp to ipsilesional MOs/MOp/SSp-ll/SSp-ul/SSs cortex pathway for large/small strokes at day one and 28 post stroke compared with control. Significant differences between the groups are shown as *p ≤ 0.05, **p ≤ 0.01, and ***p ≤ 0.001, respectively

We analyzed the structural connectivity of the transhemispheric pathway, the contralesional DORsm to the stroke-affected sensorimotor cortex (Fig. 4a). At 4 weeks post stroke, qualitative fiber tracking results indicated that in the small strokes group, fewer fibers remained, while the large stroke group was similar to the control group (Fig. 4b). Fractional anisotropy (FA), a diffusion measure of microstructural integrity, developed differently over time, depending on the stroke size (at day 28 *p* = 0.009). While the small strokes showed no significant change, FA was significantly reduced in the large strokes group compared with the controls at day 21 as well as day 28 (*p* = 0.004 and *p* = 0.034) (Fig. 4e). Similar to the findings for the ipsilesional thalamocortical pathways, the overall transhemispheric thalamocortical connectivity was decreased for both stroke groups at day 1 (*p* < 0.001). While the structural connectivity remained reduced at day 28 in the small strokes compared with the controls (*p* < 0.001), it was increased in the large group (*p* = 0.023) and back to the level of control mice (*p* = 0.496) (Fig. 4f). According to the detailed analysis of the top 5 transhemispheric thalamocortical targets, the connectivity of several pathways was decreased at 1 day post stroke compared with control (e.g., SSp-ul and SSs). Importantly, only the DORsm to SSp-ul pathway was significantly increased in the large stroke group (*p* = 0.040).

### Increased Structural Connectivity Between Homotopic Cortical Areas

Next to the thalamocortical pathways, we analyzed the transhemispheric homotopic connectivity of the primary and secondary sensorimotor cortex regions affected in both stroke groups (Fig. 4c). The first indication of a differential structural re-arrangement was found for the MOp regions, which increased stronger in the large strokes group over time (comparison of linear regressions, *p* = 0.049) (Fig. 4d). The sum of all fiber tracks from the contralesional MOp to the ipsilesional MOp, MOs, SSp-ll, SSp-ul, and SSs showed a decrease at day 1 only between the controls and the large strokes group (*p* = 0.001) but not the small strokes group. Notably, transhemispheric structural connectivity was significantly increased in the large strokes group from day 1 to 28 (*p* < 0.001) back to control levels (*p* = 0.053) (Fig. 4g, h). The detected increase was mainly due to higher levels of structural connectivity in MOs and SSp-ll, a region affected by the stroke throughout the 4-week observation period (*p* = 0.030).

## Discussion

Here, we have characterized the impact of cortical brain lesions on the integrity of sensorimotor fiber tracts and spontaneous behavioral improvement over 4 weeks in adult mice. The study was motivated by previous reports about spontaneous recovery after stroke, which requires different brain areas for efficient remodeling, depending on the lesion size. While small strokes may recruit peri-infarct tissue for functional remapping, large strokes require unaffected regions at more distant sites such as the contralesional hemisphere for recovery of function [18]. Furthermore, our focus was on cortical stroke lesions in the somatosensory and motor areas because of the large body of evidence of post-stroke plasticity related to the altered local dendritic spine turnover and axonal sprouting as well as short-range to long-range inter- and intracortical connectivity [19–21].

### Lesion Size and Location

In order to efficiently compare the lesion size and location, we applied a detailed atlas registration workflow [22, 23], which provided unbiased and automated lesion mapping, connectivity analysis, and histology. For the stroke segmentation, we used the T2-weighted MRI (T2WI), which according to a recent meta-analysis, serves as an effective noninvasive assessment of infarct size during the first 2 weeks after the onset of ischemia [24]. Different to other stroke models, in photothrombotic stroke, the vasogenic edema which relates to the strong hyperintense signal on T2WI, was reported to be quickly resolved during the first 2 weeks after stroke and replaced by a hypointense cavity [25]. In our experiments, the clearance of the vasogenic edema was delayed in larger strokes. The final lesion size was determined using GFAP immunostaining, an established method in photothrombotic stroke research, to visualize activated astrocytes surrounding the necrotic stroke core as glial scar [25, 26]. In order to challenge the endogenous mechanisms after stroke, we applied two photothrombotic stroke protocols resulting in a group of mice with small and large strokes, respectively. Initially, the two groups were separated by a lesion size factor of 1.8 and significant larger involvement of the primary and secondary motor cortex, which increased to a lesion size factor of 2.7 at 4 weeks after stroke due to a more persistent lesion area in the motor cortex of the large strokes group. In summary, large T2WI lesions with large involvement of the motor cortex on day 1 correlated with a large deficit but did not predict reliably the functional outcome. That finding is in line with the ongoing debate in clinical studies that brain lesion profiles (which is a combination of size and location) rather than the absolute lesion size alone correlate with the outcome [4]. Some studies showed that initial MRI-based lesion size is negatively correlated with functional outcome [27], and an outcome surrogate [28] with the limitation that the prognostic relevance of heterogeneous clinical stroke locations relates to brain lesions with different types and levels of functional impairment [29]. Instead, in our study, the functional deficit on day 3 was found to be a strong indication of the lesion size 4 weeks after stroke. However, the chronic lesion size itself was no indicator of the functional outcome. These results suggest that there is a differential compensatory, or functional recovery process is initiated in the large compared with the small strokes group, which masks the detrimental effect of the larger stroke lesion over time.

### Secondary Neurodegeneration

We studied secondary neurodegeneration (SND) as one crucial histopathological mechanism after stroke, which initiates ongoing cell death selectively in non-ischemic brain areas with synaptic connections to the primary lesion site and correlates with the functional deficit and outcome [9]. In line with previous results [30–32], we detected SND based on the increased accumulation of astrogliosis (GFAP+ cells) and microglia/macrophages (Iba1+ cells) accompanied with more iron deposition, which was selective to the DORsm part of the thalamus incorporating, e.g., the ventroposterior thalamic nucleus (VPN). Besides, the DTI analysis revealed elevated radial diffusivity, a marker of axonal damage [33], selectively in large strokes’ DORsm but not DORpm at 4 weeks post stroke. Based on the DTI fiber tracking for the DORsm, we found SND of thalamocortical tracts in the large strokes group with the most substantial effect on the upper and lower limp representation. However, the large strokes group behavioral outcome was the same as the small group, pointing towards an additional effect triggered selectively in more extensive lesions.

### Transhemispheric Plasticity

Post-stroke reorganization of motor representation is related to thalamo-cortical loops, a redundant system to integrate sensory and motor signals [34]. In the acute phase after stroke, rapid changes in sensory processing occur within the peri-infarct zone, which extend to somatotopic regions of the unaffected hemisphere related to enhanced sensory responses [35]. In our studies of the chronic phase after stroke, we found enhanced transhemispheric rerouting, indicated by elevated FA levels and structural connectivity equal to control levels in the larger strokes only. Increased activation of the unaffected motor cortex, mediated by disinhibition, and the formation of novel, atypical connections with the ipsilesional hemisphere have been implicated in stroke recovery [36, 37]. In our data set, FA levels in the ipsilesional MOp increased stronger in the larger strokes accompanied by increased interhemispheric motor output reaching pre-stroke levels. Similarly, increased interhemispheric motor cortex connectivity and higher FA in the transcallosal motor fibers were related in a clinical study to less impairment [38]. Furthermore, it was hypothesized that the lesion volume and the amount of spared tissue in the ipsilesional hemisphere determine the beneficial or detrimental role of the contralesional hemisphere [39]. In support of our data, functional studies have shown that the recruitment of contralesional cortex depends mainly on whether the primary motor cortex (M1) was lesioned [40] and the (activity of the) healthy hemisphere is necessary for recovery when the lesion involves large parts of the motor area (as it was the case for the large strokes group) [41, 42]. Taken together, our results add a new level of corticortical and thalamocortical network adaption to the previously described plasticity changes in ipsilesional pathways and axonal outgrowth of midline-crossing contralesional corticospinal fibers [18, 43, 44]. By comparing two different lesion sizes, we provide evidence for an additional transhemispheric plasticity mechanism of subcortical areas that contributes in large strokes to spontaneous functional improvement in large strokes.

### Limitations

The major limitations of our study are the sample size and the type of stroke model. The sample size is relatively small; however, all mice were scanned longitudinally so that each mouse served as its own control including a baseline scan before the stroke. We have used photothrombotic stroke, which simultaneously disrupts endothelial integrity with a rapidly progressing ischemic infarction and cell death in a relatively small cortical volume. Different from other animal models of stroke, which affect simultaneously cortical and subcortical areas including a more pronounced penumbra, however, requires a more invasive surgery such as middle cerebral artery occlusion [45]. Nevertheless, photothrombosis is an established model to study small stroke lesions with comparable size to human stroke in a defined area of interest [21]. Photothrombotic stroke allowed us to study the dynamic changes in somatosensory pathways without conflicting additional lesions in subcortical areas which would also have a direct effect on these sensorimotor pathways. Notably, the resulting behavioral deficit is small and only detectable with fine grade measures. The experimental time window of 4 weeks was selected based on our previous spontaneous recovery study in mice and because it corresponds to the highest period of circuit plasticity in patients (approx. 3 months after stroke) [46, 47]. However, we cannot exclude that with an even more detailed kinematic analysis, the behavioral deficit of large and maybe also small strokes would last beyond the critical 3 to 4 weeks time point. Furthermore, other tests, such as skilled reaching combined with an automated kinematic analysis, could be helpful to discriminate compensation vs. restitution related to the circuit reorganization.

For the primary readout, the structural connectivity using in vivo DTI, we have applied a protocol with the highest spatial resolution described in the literature to date. That was necessary to achieve registration with the mouse brain atlas while keeping the influence of interpolation minimal. The whole-brain DTI connectivity analysis provided structural networks with a very high correlation to viral tracing experiments [17]. In line with previous studies, which reported a high correlation of the myelin microstructure to DTI, such as high FA values linked to high myelin density [48, 49], we found a strong correlation of fiber tracking of the thalamocortical network (DORsm to cortex) with viral tracing data [17]. Notably, such correlation does not imply 3D anatomical correspondence, which could be different as others reported [50] and would need a more in-depth comparison of DTI to viral tracing in 3D such as cleared brain tissue [51]. Likewise, our DTI analysis could not discriminate between modulation of myelin composition, which is remodeling existing pathways to enhance connectivity, the sprouting of new fiber tracts or an increase in axon diameter [52]. One potential underlying mechanism, the neuronal activity-driven myelin plasticity [53], would need additional studies, e.g., utilizing simultaneous local field potential or resting-state functional MRI (rs-fMRI) studies [13, 54]. In this line, we need to stress that DTI contains no directionality, and the result for the fibers between two selected regions (e.g., the DORsm and the SSp-ul) did not discriminate how often and how many regions were passed in between.

Finally, our study is the first to show dynamic changes of the somato-motor structural connectivity in experimental stroke. Thus, the results need replication in other stroke models and multi-center studies. Nevertheless, our data present the necessary first step for the development of connectivity measures as a novel biomarker of functional outcome after stroke.

## Materials and Methods

### Experimental Study Design and Animals

Animals were housed in individually ventilated cages under 12 h light/12 h darkness cycle with access to water and food ad libitum. In total, *n* = 18 adult C57Bl/6J mice (8–10 weeks old at the start of the experiments, stock #000664, The Jackson Laboratory, USA) were used in an exploratory imaging study, for which only prior knowledge and power analysis for additional behavioral testing existed (*n* = 4 mice necessary to discriminate stroke from sham mice). Two different photothrombotic stroke protocols were applied, and *n* = 5 mice did not survive the full experimental protocol or needed to be sacrificed according to the animal protocol score sheet. Therefore, *n* = 5 small and *n* = 4 large strokes as well as *n* = 4 sham mice were included in the study. From the *n* = 4 sham mice MRI data, which were measured repetitively 4 times (due to technical reasons, not 5 times as per protocol), a control group was generated for the DTI analysis.

The mice were allocated to experimental groups randomly and assigned a unique study ID. All experimenters were blinded against the experimental group (small/large stroke, sham) during the data recording as well as the primary data analysis (such as the video evaluation of the behavior tests). The project and all related experimental data were managed using an electronic research database [55]. The experimental design is shown in Supplementary Material, Fig. 4. Animals were habituated and trained to perform the behavioral tests three times during the week before the stroke. Three days before stroke surgery, the baseline behavior was recorded. After stroke, repetitive behavior tests and MR imaging were performed at days 1, 3, 7, 14, 21, and 28 followed by animal perfusion and brain tissue preparation for histology.

### Statistics and Data Visualization

All statistical tests and data plotting were performed with Prism (macOS version 8.2.1, www.graphpad.com). The data is plotted as box plot (5–95% percentile), bar graph (mean ± sd), and line graph (mean ± sem). The significance levels are *p < 0.05, **p < 0.01, and ***p < 0.001. Normality was checked with the Kolmogorov-Smirnov test in order to decide for parametric or non-parametric statistical tests. The lesion size and location for both, MRI and histology, and the iron accumulation were compared using a two-tailed *t* test—in case of multiple comparisons corrected using the Holm-Sidak method. The DTI parameters (FA, RD, AD, and MD) and the behavioral test results (rotating beam, hindlimb drop, and cylinder test paw drag) were compared over time per group and between the groups (small strokes, large strokes, control) using a mixed-effects analysis with Bonferroni correction for multiple comparisons. A repeated-measures ANOVA with Tukey correction was used for the behavioral tests grid walk, mNDS, and rotating beam (distance/speed). Multiple comparisons were only reported when the interaction between the groups and/or over time was significant (*p* < 0.05). The fiber tracking results were compared using a Kruskal-Wallis test followed by a Dunn’s multiple comparison test, and unpaired groups were compared using the two-tailed Mann-Whitney test. For the hierarchical cluster analysis using Ward’s method of the lesion area data, IBM SPSS Statistics (macOS version 25, https://www.ibm.com/de-de/analytics/spss-statistics-software) was used.

## Electronic supplementary material

ESM 1 (PDF 616 kb)

## Data Availability

The datasets generated during and/or analyzed during the current study are available from the corresponding author on reasonable request. The MRI processing pipeline AIDAmri is included in the published article Pallast et al. [22] and available together with the custom version of the Allen Mouse Brain Atlas from Github (https://github.com/maswendt/).

## References

[1] Katan M, Luft A (2018). Global burden of stroke. Semin Neurol.

[2] Minnerup J, Wersching H, Schilling M, Schabitz WR (2014). Analysis of early phase and subsequent phase III stroke studies of neuroprotectants: outcomes and predictors for success. Exp Transl Stroke Med.

[3] Alexander LD, Black SE, Gao F, Szilagyi G, Danells CJ, McIlroy WE (2010). Correlating lesion size and location to deficits after ischemic stroke: the influence of accounting for altered peri-necrotic tissue and incidental silent infarcts. Behav Brain Funct.

[4] Chen CL, Tang FT, Chen HC, Chung CY, Wong MK (2000). Brain lesion size and location: effects on motor recovery and functional outcome in stroke patients. Arch Phys Med Rehabil.

[5] Pineiro R, Pendlebury ST, Smith S, Flitney D, Blamire AM, Styles P, Matthews PM (2000). Relating MRI changes to motor deficit after ischemic stroke by segmentation of functional motor pathways. Stroke..

[6] Sommer CJ (2017). Ischemic stroke: experimental models and reality. Acta Neuropathol.

[7] Overman JJ, Clarkson AN, Wanner IB, Overman WT, Eckstein I, Maguire JL (2012). A role for ephrin-A5 in axonal sprouting, recovery, and activity-dependent plasticity after stroke. Proc Natl Acad Sci U S A.

[8] Brown CE, Li P, Boyd JD, Delaney KR, Murphy TH (2007). Extensive turnover of dendritic spines and vascular remodeling in cortical tissues recovering from stroke. J Neurosci.

[9] Zhang J, Zhang Y, Xing S, Liang Z, Zeng J (2012). Secondary neurodegeneration in remote regions after focal cerebral infarction: a new target for stroke management?. Stroke..

[10] Hiltunen M, Jolkkonen J. Letter by Hiltunen and Jolkkonen regarding article, "secondary neurodegeneration in remote regions after focal cerebral infarction: a new target for stroke management?". Stroke. 2012;43(9):e96; author reply e7. 10.1161/STROKEAHA.112.665752. 10.1161/STROKEAHA.112.665752 22927461

[11] Hoehn-Berlage M, Eis M, Back T, Kohno K, Yamashita K (1995). Changes of relaxation times (T1, T2) and apparent diffusion coefficient after permanent middle cerebral artery occlusion in the rat: temporal evolution, regional extent, and comparison with histology. Magn Reson Med.

[12] Jiang Q, Zhang ZG, Chopp M (2010). MRI evaluation of white matter recovery after brain injury. Stroke..

[13] Dijkhuizen RM, van der Marel K, Otte WM, Hoff EI, van der Zijden JP, van der Toorn A, van Meer M (2012). Functional MRI and diffusion tensor imaging of brain reorganization after experimental stroke. Transl Stroke Res.

[14] Granziera C, D'Arceuil H, Zai L, Magistretti PJ, Sorensen AG, de Crespigny AJ (2007). Long-term monitoring of post-stroke plasticity after transient cerebral ischemia in mice using in vivo and ex vivo diffusion tensor MRI. Open Neuroimaging J.

[15] Obenaus A, Ashwal S (2012). Neuroimaging of stroke and ischemia in animal models. Transl Stroke Res.

[16] Lein ES, Hawrylycz MJ, Ao N, Ayres M, Bensinger A, Bernard A, Boe AF, Boguski MS, Brockway KS, Byrnes EJ, Chen L, Chen L, Chen TM, Chin MC, Chong J, Crook BE, Czaplinska A, Dang CN, Datta S, Dee NR, Desaki AL, Desta T, Diep E, Dolbeare TA, Donelan MJ, Dong HW, Dougherty JG, Duncan BJ, Ebbert AJ, Eichele G, Estin LK, Faber C, Facer BA, Fields R, Fischer SR, Fliss TP, Frensley C, Gates SN, Glattfelder KJ, Halverson KR, Hart MR, Hohmann JG, Howell MP, Jeung DP, Johnson RA, Karr PT, Kawal R, Kidney JM, Knapik RH, Kuan CL, Lake JH, Laramee AR, Larsen KD, Lau C, Lemon TA, Liang AJ, Liu Y, Luong LT, Michaels J, Morgan JJ, Morgan RJ, Mortrud MT, Mosqueda NF, Ng LL, Ng R, Orta GJ, Overly CC, Pak TH, Parry SE, Pathak SD, Pearson OC, Puchalski RB, Riley ZL, Rockett HR, Rowland SA, Royall JJ, Ruiz MJ, Sarno NR, Schaffnit K, Shapovalova NV, Sivisay T, Slaughterbeck CR, Smith SC, Smith KA, Smith BI, Sodt AJ, Stewart NN, Stumpf KR, Sunkin SM, Sutram M, Tam A, Teemer CD, Thaller C, Thompson CL, Varnam LR, Visel A, Whitlock RM, Wohnoutka PE, Wolkey CK, Wong VY, Wood M, Yaylaoglu MB, Young RC, Youngstrom BL, Yuan XF, Zhang B, Zwingman TA, Jones AR (2007). Genome-wide atlas of gene expression in the adult mouse brain. Nature..

[17] Oh SW, Harris JA, Ng L, Winslow B, Cain N, Mihalas S, Wang Q, Lau C, Kuan L, Henry AM, Mortrud MT, Ouellette B, Nguyen TN, Sorensen SA, Slaughterbeck CR, Wakeman W, Li Y, Feng D, Ho A, Nicholas E, Hirokawa KE, Bohn P, Joines KM, Peng H, Hawrylycz MJ, Phillips JW, Hohmann JG, Wohnoutka P, Gerfen CR, Koch C, Bernard A, Dang C, Jones AR, Zeng H (2014). A mesoscale connectome of the mouse brain. Nature..

[18] Murphy TH, Corbett D (2009). Plasticity during stroke recovery: from synapse to behaviour. Nat Rev Neurosci.

[19] Brown CE, Wong C, Murphy TH (2008). Rapid morphologic plasticity of peri-infarct dendritic spines after focal ischemic stroke. Stroke..

[20] Nudo RJ (2007). Postinfarct cortical plasticity and behavioral recovery. Stroke..

[21] Carmichael ST (2003). Plasticity of cortical projections after stroke. Neuroscientist..

[22] Pallast N, Diedenhofen M, Blaschke S, Wieters F, Wiedermann D, Hoehn M, et al. Processing pipeline for atlas-based imaging data analysis of structural and functional mouse brain MRI (AIDAmri). Front Neuroinform. 2019;13. 10.3389/fninf.2019.00042. 10.3389/fninf.2019.00042 PMC6559195 31231202

[23] Pallast N, Wieters F, Fink GR, Aswendt M (2019). Atlas-based imaging data analysis tool for quantitative mouse brain histology (AIDAhisto). J Neurosci Methods.

[24] Milidonis X, Marshall I, Macleod MR, Sena ES (2015). Magnetic resonance imaging in experimental stroke and comparison with histology: systematic review and meta-analysis. Stroke.

[25] Li H, Zhang N, Lin HY, Yu Y, Cai QY, Ma L, Ding S (2014). Histological, cellular and behavioral assessments of stroke outcomes after photothrombosis-induced ischemia in adult mice. BMC Neurosci.

[26] Porritt MJ, Andersson HC, Hou L, Nilsson A, Pekna M, Pekny M, Nilsson M (2012). Photothrombosis-induced infarction of the mouse cerebral cortex is not affected by the Nrf2-activator sulforaphane. PLoS One.

[27] Schiemanck SK, Kwakkel G, Post MW, Prevo AJ (2006). Predictive value of ischemic lesion volume assessed with magnetic resonance imaging for neurological deficits and functional outcome poststroke: a critical review of the literature. Neurorehabil Neural Repair.

[28] Farr TD, Wegener S (2010). Use of magnetic resonance imaging to predict outcome after stroke: a review of experimental and clinical evidence. J Cereb Blood Flow Metab.

[29] Cheng B, Forkert ND, Zavaglia M, Hilgetag CC, Golsari A, Siemonsen S, Fiehler J, Pedraza S, Puig J, Cho TH, Alawneh J, Baron JC, Ostergaard L, Gerloff C, Thomalla G (2014). Influence of stroke infarct location on functional outcome measured by the modified Rankin Scale. Stroke..

[30] Schroeter M, Zickler P, Denhardt DT, Hartung HP, Jander S (2006). Increased thalamic neurodegeneration following ischaemic cortical stroke in osteopontin-deficient mice. Brain.

[31] Justicia C, Ramos-Cabrer P, Hoehn M (2008). MRI detection of secondary damage after stroke: chronic iron accumulation in the thalamus of the rat brain. Stroke..

[32] Dihne M, Grommes C, Lutzenburg M, Witte OW, Block F (2002). Different mechanisms of secondary neuronal damage in thalamic nuclei after focal cerebral ischemia in rats. Stroke..

[33] Song SK, Sun SW, Ramsbottom MJ, Chang C, Russell J, Cross AH (2002). Dysmyelination revealed through MRI as increased radial (but unchanged axial) diffusion of water. NeuroImage..

[34] Balbinot G, Schuch CP (2018). Compensatory relearning following stroke: cellular and plasticity mechanisms in rodents. Front Neurosci.

[35] Mohajerani MH, Aminoltejari K, Murphy TH (2011). Targeted mini-strokes produce changes in interhemispheric sensory signal processing that are indicative of disinhibition within minutes. Proc Natl Acad Sci U S A.

[36] Dancause N (2006). Vicarious function of remote cortex following stroke: recent evidence from human and animal studies. Neuroscientist..

[37] Rehme AK, Fink GR, von Cramon DY, Grefkes C (2011). The role of the contralesional motor cortex for motor recovery in the early days after stroke assessed with longitudinal FMRI. Cereb Cortex.

[38] Asrican B, Augustine GJ, Berglund K, Chen S, Chow N, Deisseroth K, Feng G, Gloss B, Hira R, Hoffmann C, Kasai H, Katarya M, Kim J, Kudolo J, Lee LM, Lo SQ, Mancuso J, Matsuzaki M, Nakajima R, Qiu L, Tan G, Tang Y, Ting JT, Tsuda S, Wen L, Zhang X, Zhao S (2013). Next-generation transgenic mice for optogenetic analysis of neural circuits. Front Neural Circuits.

[39] Alia C, Spalletti C, Lai S, Panarese A, Lamola G, Bertolucci F, Vallone F, di Garbo A, Chisari C, Micera S, Caleo M (2017). Neuroplastic changes following brain ischemia and their contribution to stroke recovery: novel approaches in neurorehabilitation. Front Cell Neurosci.

[40] Feydy A, Carlier R, Roby-Brami A, Bussel B, Cazalis F, Pierot L, Burnod Y, Maier MA (2002). Longitudinal study of motor recovery after stroke: recruitment and focusing of brain activation. Stroke..

[41] Di Pino G, Pellegrino G, Assenza G, Capone F, Ferreri F, Formica D (2014). Modulation of brain plasticity in stroke: a novel model for neurorehabilitation. Nat Rev Neurol.

[42] Biernaskie J, Szymanska A, Windle V, Corbett D (2005). Bi-hemispheric contribution to functional motor recovery of the affected forelimb following focal ischemic brain injury in rats. Eur J Neurosci.

[43] Wahl AS, Omlor W, Rubio JC, Chen JL, Zheng H, Schroter A (2014). Neuronal repair. Asynchronous therapy restores motor control by rewiring of the rat corticospinal tract after stroke. Science..

[44] Liu Z, Li Y, Zhang X, Savant-Bhonsale S, Chopp M (2008). Contralesional axonal remodeling of the corticospinal system in adult rats after stroke and bone marrow stromal cell treatment. Stroke..

[45] Carmichael ST. Rodent models of focal stroke: size, mechanism, and purpose. NeuroRx. 2005;2(3):396–409. 10.1602/neurorx.2.3.396. 10.1602/neurorx.2.3.396 PMC1144484 16389304

[46] Zeiler SR, Krakauer JW (2013). The interaction between training and plasticity in the poststroke brain. Curr Opin Neurol.

[47] Ito M, Aswendt M, Lee AG, Ishizaka S, Cao Z, Wang EH, Levy SL, Smerin DL, McNab J, Zeineh M, Leuze C, Goubran M, Cheng MY, Steinberg GK (2018). RNA-sequencing analysis revealed a distinct motor cortex transcriptome in spontaneously recovered mice after stroke. Stroke..

[48] Harsan LA, David C, Reisert M, Schnell S, Hennig J, von Elverfeldt D (2013). Mapping remodeling of thalamocortical projections in the living reeler mouse brain by diffusion tractography. Proc Natl Acad Sci U S A.

[49] Seehaus A, Roebroeck A, Bastiani M, Fonseca L, Bratzke H, Lori N, Vilanova A, Goebel R, Galuske R (2015). Histological validation of high-resolution DTI in human post mortem tissue. Front Neuroanat.

[50] Calabrese E, Badea A, Cofer G, Qi Y, Johnson GA (2015). A diffusion MRI tractography connectome of the mouse brain and comparison with neuronal tracer data. Cereb Cortex.

[51] Goubran M, Leuze C, Hsueh B, Aswendt M, Ye L, Tian Q, Cheng MY, Crow A, Steinberg GK, McNab J, Deisseroth K, Zeineh M (2019). Multimodal image registration and connectivity analysis for integration of connectomic data from microscopy to MRI. Nat Commun.

[52] Dyrby TB, Sogaard LV, Hall MG, Ptito M, Alexander DC (2013). Contrast and stability of the axon diameter index from microstructure imaging with diffusion MRI. Magn Reson Med.

[53] Foster AY, Bujalka H, Emery B (2019). Axoglial interactions in myelin plasticity: evaluating the relationship between neuronal activity and oligodendrocyte dynamics. Glia..

[54] Vallone F, Lai S, Spalletti C, Panarese A, Alia C, Micera S, Caleo M, di Garbo A (2016). Post-stroke longitudinal alterations of inter-hemispheric correlation and hemispheric dominance in mouse pre-motor cortex. PLoS One.

[55] Pallast N, Wieters F, Nill M, Fink GR, Aswendt M. Cloud-based relational database for multimodal animal data. Database (Oxford). 2018;2018. doi:10.1093/database/bay124. 10.1093/database/bay124 PMC6301331 30576483

