# Supplementary material for: Lesion Size- and Location-Dependent Recruitment of Contralesional Thalamus and Motor Cortex Facilitates Recovery after Stroke in Mice
Source: Transl Stroke Res. 2020 Mar 12;12(1):87–97. doi: 10.1007/s12975-020-00802-3 (PMC7803721; doi:10.1007/s12975-020-00802-3)
Supplement: Supplementary file 1 — (PDF 616 kb) [file 12975_2020_802_MOESM1_ESM.pdf]

SUPPLEMENTARY FIGURES AND TABLES

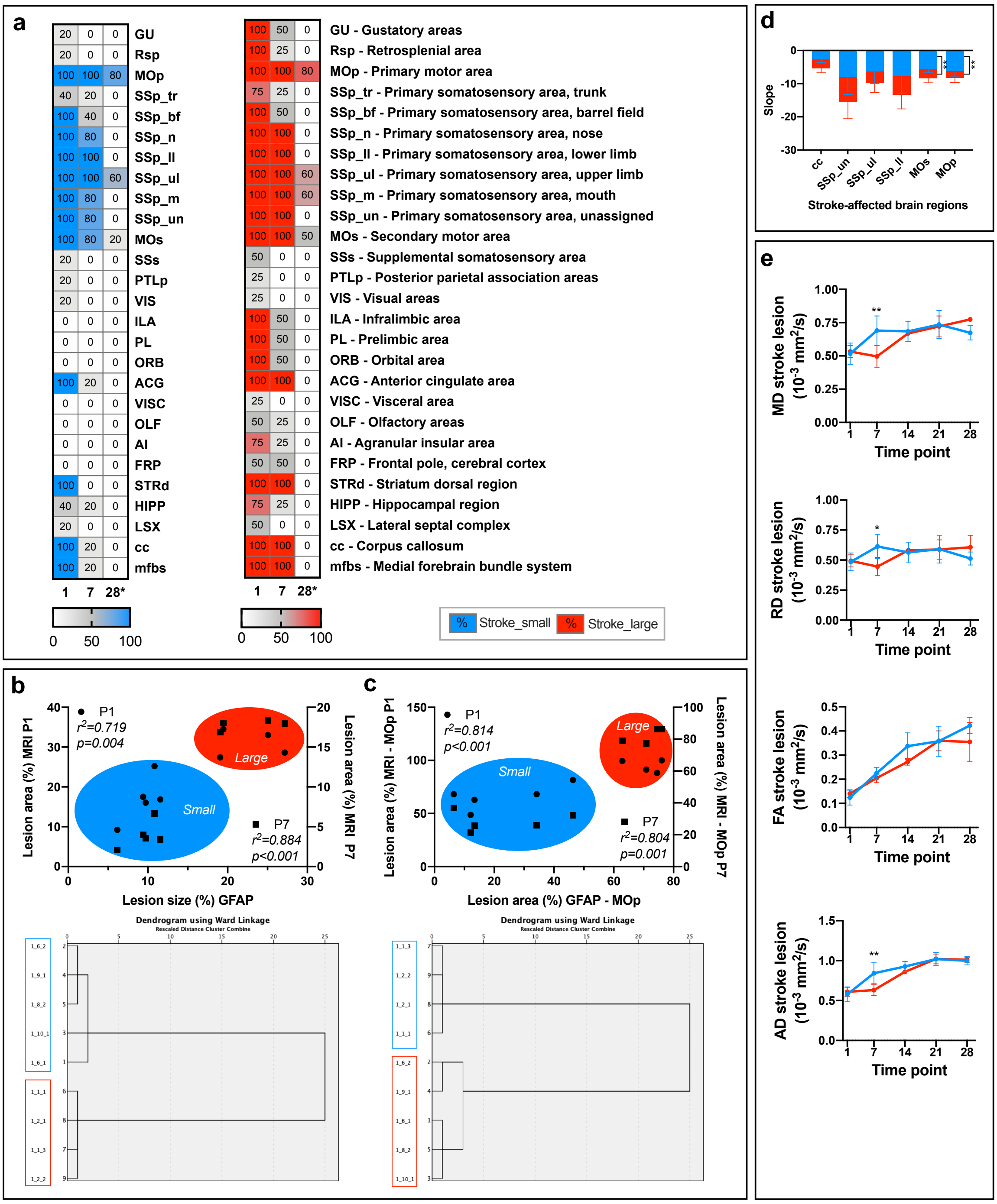

Fig. 1: Correlations of MRI with histology and additional analysis of diffusion measures. **a** Lesion mapping from in vivo and ex vivo imaging summarized as heat maps for small (blue) and large (red) strokes; color code indicates the percentage of mice in which a specific brain region was inside the stroke mask at days 1/7 (MRI) and 28* (histology), respectively. **b-c** Pearson correlation of lesion size and lesion area percentage in MOp region determined by MRI and histology, respectively. The R^2 and p-value are shown. Schematic drawings of blue and red colored areas highlighting the two distinct clusters related to the small and large strokes group, respectively. Results of the hierarchical cluster analysis with Ward's method are shown below as correlation graphs. **d** Decay in percent infarct area per region expressed as the slope between days 1 and 7 for selected brain regions. **e** Diffusion measured inside the stroke mask for the small (blue) and large (red) strokes group. Results plotted as mean diffusivity (MD), fractional anisotropy (FA), radial diffusivity (RD) and axial diffusivity (AD). Data in **d** and **e** are shown as and mean±sd. Significant differences between the groups are shown as * for $p<0.05$ and ** for $p<0.01$, respectively

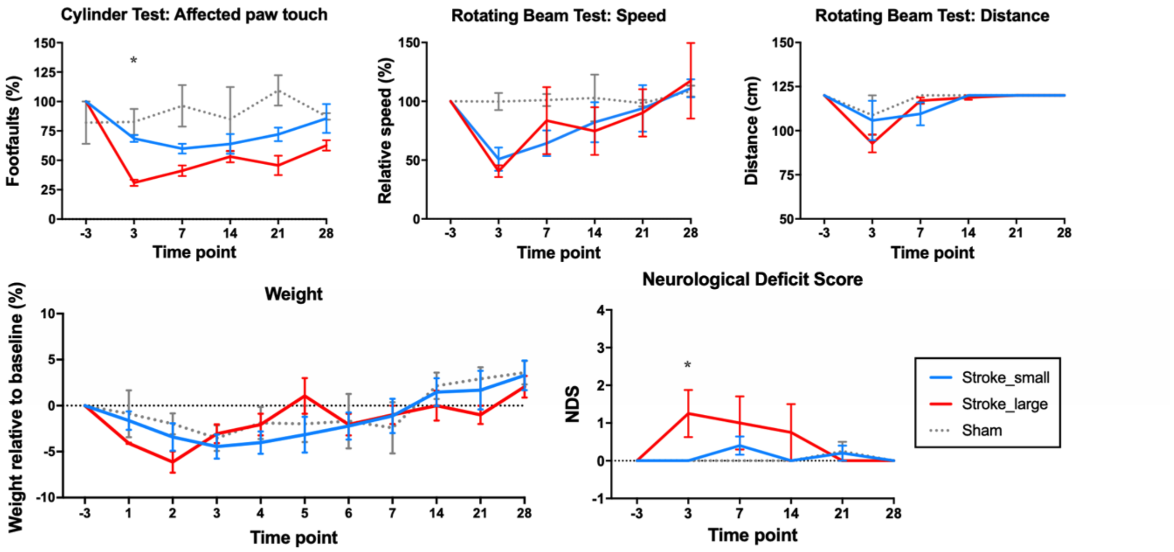

Fig. 2: Additional measures of the Cylinder, Rotating Beam Test and monitoring of weight and Neurological Deficit Score. Data in graphs are shown as mean±sem. Significant differences between the groups at the same time points are shown as * for p≤0.05

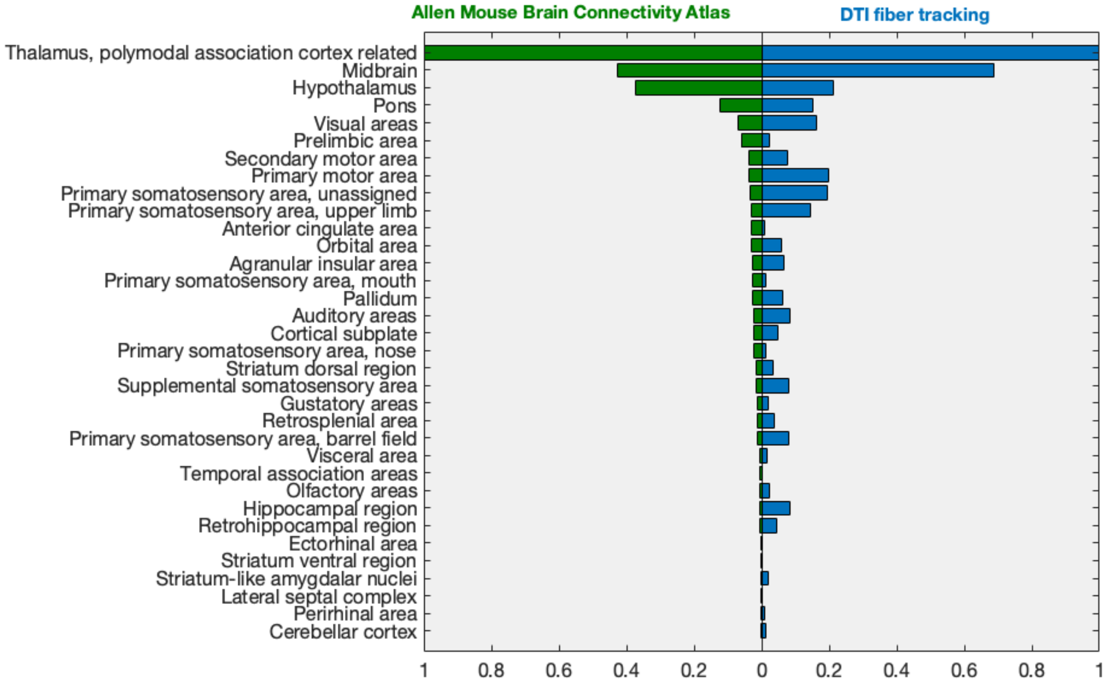

Fig. 3 Comparison of connectivity between the sensory-motor cortex related part of the thalamus (DORsm) based on the viral tracing experiments of the Allen Mouse Brain Connectivity Atlas and our DTI fiber tracking of the control group. Viral projection density from n=11 injection experiments averaged and was normalized to brain region size. Fiber density from n=4 mice measured four times averaged and normalized to brain region size. For visualization, the scale was normalized to the region with the strongest connectivity of the polymodal association related part of the thalamus (DORpm)

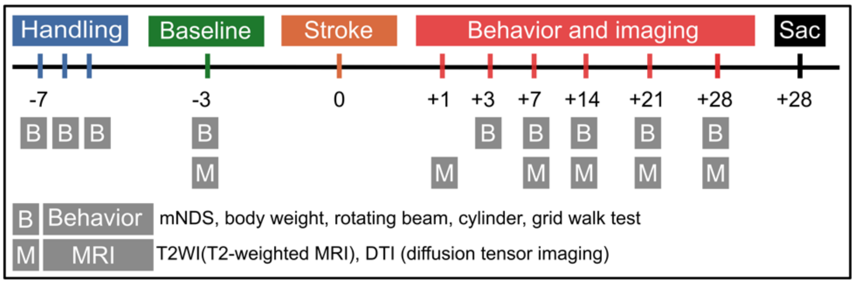

Fig. 4 Experimental design

Table 1: Pearson correlations of lesion size and location with behavior.

	(Cylinder Test) paw drags	(Grid Walk) footfaults	(Rotating Beam) hindlimb drops
**Day 1 MRI vs. day 3 behavioral deficit**			
R^2	0.434	0.499	0.465
P (two-tailed)	0.054	0.033*	0.043*
**Day 1 MRI %MOp vs. day 3 behavioral deficit**			
R^2	0.090	0.5459	0.6321
P (two-tailed)	0.432	0.023*	0.010*
**Day 1 MRI vs. day 28 behavioral outcome**			
R^2	0.361	0.006	0.043
P (two-tailed)	0.087	0.838	0.593
**Day 1 MRI %MOp vs. day 28 behavioral outcome**			
R^2	0.479	0.001	0.072
P (two-tailed)	0.039*	0.9386	0.483
**Day 7 MRI vs. day 28 behavioral outcome**			
R squared	0.384	0.028*	0.048*
P (two-tailed)	0.075	0.670	0.570
**Day 7 MRI %MOp vs. day 28 behavioral outcome**			
R^2	0.673	0.242	0.156
P (two-tailed)	0.047*	0.530	0.688
**Day 28 histology vs. day 28 behavioral outcome**			
R^2	0.312	0.0065	0.036
P (two-tailed)	0.118	0.837	0.624
**Day 28 histology %MOp vs. day 28 behavioral outcome**			
R squared	0.608	0.017	0.194
P (two-tailed)	0.083	0.967	0.617
**Day 3 behavior vs. day 28 histology**			
R^2	0.142	0.801	0.774
P (two-tailed)	0.317	0.001**	0.002**
**Day3 behavior vs. day 28 histology %MOp**			
R^2	0.461	0.644	0.624
P (two-tailed)	0.212	0.061	0.073

SUPPLEMENTARY MATERIALS AND METHODS

Photothrombosis

The photothrombosis protocol to induce cortical lesions was applied as reported previously [1] with a variation in the amount of injected photosensitive Rose Bengal and different intensities of laser radiation. Briefly, the mice were anesthetized with 3-4% Isoflurane in oxygen and placed in a stereotactic frame (#504926, WPI, Germany). The surface of the head was disinfected with povidone-iodine (Betaisodona, Mundipharma, Germany) and an incision along the midline from the eye level to the neck (~1.5cm) was made. The periosteum was retracted, and the surface of the skull was cleaned with PBS and a cotton swab. A laser (MGL-FN-561 nm, CNI, China), fixed on the stereotactic frame, was pointed on Bregma and the laser spot was moved to the coordinates of the primary motor cortex (M/L: 2.00 mm and A/P: 0.00 mm). To induce a small stroke, the animals received an intraperitoneal (i.p.) injection of 1000 µg of the photosensitive dye Rose Bengal (#A17053, Alfa Aesar, Germany). After the dye was allowed to distribute in the whole organism, the laser was projected through the intact skull for 15 min with a laser intensity of 35 mW. Laser intensity was calibrated before the experiment using a power meter (PM121D, ThorLabs, Germany). The large stroke animals received 1500 µg Rose Bengal and were radiated with a laser intensity of 50 mW for 15min. Sham-surgery was conducted by the same procedures, including the injection of 1000 µg Bengal Rose but without the radiation. The wound was closed, and the animals were allowed to recover in a pre-warmed heating chamber (V1200, MediHeat). The analgesia included treatment with 1 mg/ml Tramadol (#100040, Grünenthal, Germany) for 3 days before and after the surgery in the drinking water and intraoperative i.p. injection of 4 mg/kg Caprofen (Rimadyl, Pfizer, Germany).

Behavioral testing and post-stroke monitoring

We used a battery of three different behavioral tests, i.e., the Rotating Beam, Grid Walk, and Cylinder Test, which have been described in the literature to show robustly sensorimotor deficits as well as recovery for up to four weeks post stroke [2, 3]. Motor coordination and balance were monitored using the Rotating Beam Test, a modification of the balance beam, as described elsewhere [2]. Briefly, the mice were trained to walk over a 120 cm long beam which rotates at 6 rpm. For four sequential trials (not one after the other), the time, distance, and number of hindlimb drops were evaluated. The trial was stopped when the mouse turned around or dropped (which happens in rare cases in the first week after stroke). A drop was assigned 60s (penalty) time. Results from the four trials were further used for the group comparison.

Next to the rotating beam walking experiment, the ability of the mice to grasp a thin wire of a metal mesh was assessed with the Grid Walk Test [4]. The mice can freely explore for up to 5 min the 30 cm large metal square grid (mesh size 12.7x12.7 mm, 1.05 mm diameter) which is placed in 50 cm height (to prevent the mice from simply escaping the arena by jumping down on the table). We assessed the

total number of footsteps and footfaults (i.e., when the palm slips through the grid or when the palm is used to rest the paw at the level of the wrist on the grid) via video anlysis. The ratio of footfaults is calculated with number of footfaults/ (number of footfaults + non-footfault steps)*100).

The setup for the Cylinder Test was adapted from [5]: a transparent plastic cylinder with 10 cm diameter and 15 cm height was video-taped through a transparent plate and a mirror from below. For the analysis, we applied the frame-by-frame strategy by Roome et al. [6], which allows a distinction between the number of paw touches at the cylinder wall and the subsequent paw drags per forelimb. Paw-dragging behavior is distinct from regular normal paw touches and includes situations when the paw will slowly fall away from the wall with slight tremor or the mouse drags its paw against the cylinder wall but does not release it entirely before dismounting. Paw-drags are expressed as a percentage of paw-drags per total number of paw touches during a session. Touches resulting in a paw-drag count as a paw-drag and a touch simultaneously.

Post-surgery monitoring included a visual inspection, weighing, and modified neurological deficit scoring (mNDS) for every day and once a week from 7 days post-surgery. The mNDS was adapted from [7] and includes measures of general deficits (appearance of the eyes and the fur, spontaneous movement, epileptic behavior) and focal deficits (whisker response, body/forelimb symmetry, circling behavior, gait).

MRI acquisition

The MRI data were acquired at the Max Planck Institute for Metabolism Research, Cologne, using a 94/20USR BioSpec Bruker system (Bruker, BioSpin, Ettlingen, Germany) equipped with a cryo-coil, the B-GA 9S gradient system, RT-shim, and related power supplies, and operated with ParaVision (v6.0.1). Mice were anesthetized initially with Isoflurane (2-3% in 70/30 N_2/O_2) and head-fixed in an animal carrier using tooth and ear bars. Respiration, and body temperature were noninvasively monitored using an MR-compatible monitoring system (Small Animal Instruments Inc., New York, NY, USA) and displayed and recorded using a custom-made data acquisition system based on DASYLab (measX, Mönchengladbach, Germany). To maintain body temperature at 37°C, a feedback-controlled water circulation system (medres, Cologne, Germany) was used. Initially, a shimming procedure on the whole volume of interest was conducted to correct the distortions and optimize the magnetic field homogeneity. Subsequently, a high-resolution, whole-brain T2-weighted RARE sequence (T2WI) with 29 slices was acquired and the slice orientation was propagated to the DTI (20 slices) to match both scans in space before image registration. The DTI was implemented by an isotropic echo planar imaging sequence with 8 segments, 5 B0 images, and 30 gradient directions, a gradient duration of 3.5 ms, a gradient separation of 8 ms, and a max. b value of 2525 s/mm^2. We decided for the commonly used diffusion-encoding scheme of Jones30[8] to reduce background-diffusion coupling for all 30 directions. T2-weighted and DTI scans (Table S2) were sequentially acquired.

Table 2: MRI parameters.

Scan type	T_R[1] [ms]	T_E[2] [ms]	Acq. time [sec]	Voxel	Resolution [mm]	FOV[3] [mm^2]	Flip angle
**T2WI**[4]	5500	32.5	352	256 x 256 x 48	0.068 x 0.068 x 0.3	17.5 x 17.5	90°
**DTI**[5]	3000	17.5	840	128 x 128 x 20	0.141 x 0.141 x 0.4	18.0 x 18.0	90°

Abbreviations: [1]repetition time, [2]echo time, [3]field of view, [4]T2-weighted MRI, [5]Diffusion Tensor Imaging.

MRI pre-processing

The MRI data were pre-processed and associated with the Allen Mouse Brain (Reference) Atlas[9], ARA, using an in-house developed processing pipeline for Atlas-based Imaging Data Analysis (AIDAmri) written in Python 3.6 [1] and available from (https://github.com/maswendt/AIDAmri). To facilitate a transfer of image information from the high-resolution ARA with 50 µm isotropic resolution to the MRI data (up to 10-times larger voxels) without interpolation, we created a parental atlas according to ARA ontology. The atlas contains 96 brain structures mirrored at the brain midline to allow left to right region comparisons. Before the ARA was registered to the acquired data, the T2WI and DTI data were pre-processed by a bias field correction and brain extraction. The dimensionality of the DTI was reduced from 4D to 3D, and a minimum filter was applied to reduce image noise and artifacts. The ARA was first non-linearly transformed to the corresponding and pre-processed T2WI data set and subsequently transferred to the DTI data set by an affine registration. The transformed ARA information was subsequently used for processing of DTI raw data.

Quantification of lesion size and location

A semiautomated approach was adapted from [10] to create 3D stroke masks on T2-weighted MR images. Briefly, pre-processed T2WI was segmented using ITK-SNAP (version 3.6.0, www.itksnap.org, [11]) by one user to keep the subjective error in delineating the T2 hyperintense stroke area from healthy tissue. As the vasogenic/cytotoxic edema clears rapidly after photothrombosis [12] and it becomes more difficult and error-prone to distinguish the stroke lesion on T2WI, stroke masks were drawn only at day 1 and 7. The stroke masks were used to calculate the lesion volume related to the whole brain volume (from the brain-extracted T2WI) and to verify the lesion location. To generate incidence maps, the stroke masks were registered and super-positioned on the image information of the ARA template. Subsequently, a color scale was applied to the overlaid masks and the ARA annotations were automatically listed per animal.

DTI analysis and fiber tracking

To minimize the anatomical aberrations caused by respiration in the raw data, we used a stratified motion correction with FSL MCFLIRT [13]. The motion-corrected data were automatically fed into DSI-Studio

[14] and reconstructed based on the b-values of the electronically optimized protocol of Jones30 [8] with 30 gradient directions. All reconstructed data sets and the properties molecular diffusion rate (MD), the directional preference of diffusion (fractional anisotropy, FA), the diffusion rate along the main axis of diffusion (axial diffusivity, AD), and the rate of diffusion in the transverse direction (radial diffusivity, RD) were separately exported. The brain trajectory tracing is performed with deterministic streamline propagation using the Euler methods (4) and limited to total fiber count of one million fibers. Fibers shorter than 0.5 mm or longer than 12 mm were discarded and the tracking was terminated when the angle between two consecutive directions exceeded 55°. Finally, the pipeline output were connectivity matrices in which the rows and columns represent an area of the ARA and the entries indicate the number of fiber tracts between the individual ARA region. From whole-brain fiber tracking, the structural connectivity for individual seeds and targets was calculated. Outliers (upper and lower 5%) per group were excluded from further analysis.

From n=4 sham animals which were measured repetitively 4-times repetitively, a control group was generated for the DTI analysis. We verified that the DTI data acquisition for these four mice was constant, invariant and time-independent with a mean covariance $c=0.046\pm0.002$ and a mean linear correlation coefficient ρ (rho)=0.941 ± 0.034 with $p<0.001$. Furthermore, we analyzed the robustness of in vivo DTI fiber tracking (n=16: four different measurements from the four sham mice) to identify structural targets of the DORsm as a seed region compared to the viral tracing data of the Allen Mouse Brain Connectivity Atlas [15] with n=11 viral injection experiments. The virus connectivity arrays included the connectivity distribution of all linked regions and were used as a reference for direct comparison with the connectivity of DTI. In order to increase the comparability between the two methods, the data of both were normalized to the value with the highest projection density (DORsm) (Supplement Material Fig. 4).

Immunohistochemistry

To preserve brain tissue for imaging and further analysis, mice were intracardially perfused with 20 ml of phosphate buffered saline (PBS, Merck, Germany), followed by 20 ml of 4% phosphate-buffered formaldehyde (PFA, Roti-Histofix, Carl Roth, Germany). After perfusion, brains were isolated and transferred for 24 hours to a post-fixative solution containing 4% PFA at 4°C. For long term storage the brains were stored in 30% sucrose solution at 4°C. Post fixation, 20 µm coronal tissue sections were prepared using a microtome with freezing stage and the slices were stored at -20°C.

The fixed tissue was immunostained following a standard protocol. The frozen sections were thawed for 10 min at room temperature (RT) and pre-treated with sodium citrate (10 mM in PBS) for 30 min at 80°C. The sections were washed with PBS and incubated for 1h at RT in blocking solution (consisting of + 5% goat serum #S-1000, Dianova; PBS + 0.25% Triton X-100), followed by incubation with antibodies against GFAP (1:500; Agilent Cat# Z0334, RRID:AB_10013382) or Iba1 (1:500; Wako Cat# 019-19741, RRID:AB_839504) over night at 4°C.

After washing with PBS, the sections were incubated with the secondary antibodies Alexa 488 (1:500; Thermo Fisher Scientific Cat# A-21441, RRID:AB_2535859) and Cy3 (1:500; Jackson ImmunoResearch Labs Cat# 711-165-152, RRID:AB_2307443) or Cy5 (1:200; JacksonImmuoResearch Labs Cat# 711-175-152, RRID:AB_2340607) diluted in BS for 2 hrs at RT. Cell nuclei were stained using DAPI (HP20.1, Carl-Roth) for 5 min after the secondary antibodies were rinsed off. The sections were washed with PBS, covered with Fluromount G (Thermo Fisher, Germany), dried overnight at RT, and stored at 4°C.

Microscopy and data analysis

For cell quantification, images were acquired using a 20x objective with a fluorescence microscope (Evos, Thermo Fisher Scientific) for selected brain sections at fixed distance from bregma: striatum (+0.13 mm) and thalamus/hippocampus (-1.91). Images were stitched using the EVOS FL Auto Cell Imaging System Software (Thermo Fisher Scientific) software.

The lesion area was manually delineated on at least n=3 sections per mouse. These had been stained using GFAP to visualize the astrogliosis surrounding the necrotic lesion area using ImageJ (version 1.52o, http://imagej.nih.gov/ij). Lesion size was calculated using the formula: size of contralesional cortex – size of ipsilesional cortex / size of contralesional hemisphere.

For automated atlas-based cell counting on the selected brain sections (striatum and thalamus/hippocampus), we used the in-house developed AIDAhisto workflow (https://github.com/maswendt/AIDAhisto) described in detail elsewhere[16]. Briefly, similar to the atlas used for the MRI registration, a parental "histology" atlas, which was based on the 10 µm instead of 50 µm isotropic resolution ARA, was created compromising 49 brain structures mirrored at the brain midline to allow left to right region comparisons. Landmark-based registration in ImageJ was used to register the atlas with the whole brain slice image. Counting parameters were adjusted for GFAP, Iba1, and DAPI once and applied for all related images. The cell count of each brain region was summed up over all subjects.

REFERENCES

1. Pallast N, Diedenhofen M, Blaschke S, Wieters F, Wiedermann D, Hoehn M et al. Processing Pipeline for Atlas-Based Imaging Data Analysis of Structural and Functional Mouse Brain MRI (AIDAmri). Front Neuroinform. 2019;13:42. doi:10.3389/fninf.2019.00042.

2. Cheng MY, Wang EH, Woodson WJ, Wang S, Sun G, Lee AG et al. Optogenetic neuronal stimulation promotes functional recovery after stroke. Proceedings of the National Academy of Sciences of the United States of America. 2014;111(35):12913-8. doi:10.1073/pnas.1404109111.

3. Balkaya M, Krober JM, Rex A, Endres M. Assessing post-stroke behavior in mouse models of focal ischemia. J Cereb Blood Flow Metab. 2013;33(3):330-8. doi:10.1038/jcbfm.2012.185.

4. Baskin YK, Dietrich WD, Green EJ. Two effective behavioral tasks for evaluating sensorimotor dysfunction following traumatic brain injury in mice. Journal of neuroscience methods. 2003;129(1):87-93.

5. Li X, Blizzard KK, Zeng Z, DeVries AC, Hurn PD, McCullough LD. Chronic behavioral testing after focal ischemia in the mouse: functional recovery and the effects of gender. Experimental neurology. 2004;187(1):94-104. doi:10.1016/j.expneurol.2004.01.004.

6. Roome RB, Vanderluit JL. Paw-dragging: a novel, sensitive analysis of the mouse cylinder test. J Vis Exp. 2015(98):e52701. doi:10.3791/52701.

7. Chen J, Sanberg PR, Li Y, Wang L, Lu M, Willing AE et al. Intravenous administration of human umbilical cord blood reduces behavioral deficits after stroke in rats. Stroke. 2001;32(11):2682-8.

8. Skare S, Hedehus M, Moseley ME, Li TQ. Condition number as a measure of noise performance of diffusion tensor data acquisition schemes with MRI. J Magn Reson. 2000;147(2):340-52. doi:10.1006/jmre.2000.2209.

9. Lein ES, Hawrylycz MJ, Ao N, Ayres M, Bensinger A, Bernard A et al. Genome-wide atlas of gene expression in the adult mouse brain. Nature. 2007;445(7124):168-76. doi:10.1038/nature05453.

10. Ito M, Aswendt M, Lee AG, Ishizaka S, Cao Z, Wang EH et al. RNA-Sequencing Analysis Revealed a Distinct Motor Cortex Transcriptome in Spontaneously Recovered Mice After Stroke. Stroke. 2018;49(9):2191-9. doi:10.1161/STROKEAHA.118.021508.

11. Yushkevich PA, Piven J, Hazlett HC, Smith RG, Ho S, Gee JC et al. User-guided 3D active contour segmentation of anatomical structures: significantly improved efficiency and reliability. NeuroImage. 2006;31(3):1116-28. doi:10.1016/j.neuroimage.2006.01.015.

12. Li H, Zhang N, Lin HY, Yu Y, Cai QY, Ma L et al. Histological, cellular and behavioral assessments of stroke outcomes after photothrombosis-induced ischemia in adult mice. BMC Neurosci. 2014;15:58. doi:10.1186/1471-2202-15-58.

13. Jenkinson M, Beckmann CF, Behrens TE, Woolrich MW, Smith SM. Fsl. NeuroImage. 2012;62(2):782-90. doi:10.1016/j.neuroimage.2011.09.015.

14. Yeh FC, Verstynen TD, Wang Y, Fernandez-Miranda JC, Tseng WY. Deterministic diffusion fiber tracking improved by quantitative anisotropy. PloS one. 2013;8(11):e80713. doi:10.1371/journal.pone.0080713.

15. Oh SW, Harris JA, Ng L, Winslow B, Cain N, Mihalas S et al. A mesoscale connectome of the mouse brain. Nature. 2014;508(7495):207-14. doi:10.1038/nature13186.

16. Pallast N, Wieters F, Fink GR, Aswendt M. Atlas-based imaging data analysis tool for quantitative mouse brain histology (AIDAhisto). Journal of neuroscience methods. 2019;326:108394. doi:10.1016/j.jneumeth.2019.108394.
